# Endocytotic uptake of FITC-labeled anti-*H. pylori* egg yolk immunoglobulin Y in *Candida* yeast for detection of intracellular *H. pylori*

**DOI:** 10.3389/fmicb.2015.00113

**Published:** 2015-02-16

**Authors:** Parastoo Saniee, Farideh Siavoshi

**Affiliations:** Department of Microbiology, School of Biology, College of Sciences, University of Tehran, Tehran, Iran

**Keywords:** *H. pylori*, FITC-IgY-Hp, endocytosis, yeast, vacuole

## Abstract

Intracellular life of *Helicobacter pylori* inside *Candida* yeast vacuole describes the establishment of *H. pylori* in yeast as a pre-adaptation to life in human epithelial cells. IgY-Hp conjugated with fluorescein isothiocyanate (FITC) has been previously used for identification and localization of *H. pylori* inside the yeast vacuole. Here we examined whether FITC-IgY-Hp internalization into yeast follows the endocytosis pathway in yeast. Fluorescent microscopy was used to examine the entry of FITC-IgY-Hp into *Candida* yeast cells at different time intervals. The effect of low temperature, H_2_O_2_ or acetic acid on the internalization of labeled antibody was also examined. FITC-IgY-Hp internalization initiated within 0–5 min in 5–10% of yeast cells, increased to 20–40% after 30 min–1 h and reached >70% before 2 h. FITC-IgY-Hp traversed the pores of *Candida* yeast cell wall and reached the vacuole where it bound with *H. pylori* antigens. Internalization of FITC-IgY-Hp was inhibited by low temperature, H_2_O_2_ or acetic acid. It was concluded that internalization of FITC-IgY-Hp into yeast cell is a vital phenomenon and follows the endocytosis pathway. Furthermore, it was proposed that FITC-IgY-Hp internalization could be recruited for localization and identification of *H. pylori* inside the vacuole of *Candida* yeast.

## INTRODUCTION

Evolution of eukaryotic cells has been regarded as the most magnificent phenomenon in the history of life. Thousands of DNA mutations in prokaryotic cells caused major changes that led to development of today’s eukaryotes. These changes included formation of endomembrane system and internal cytoskeleton that favored engulfment of smaller prokaryotes through the mechanism of phagotrophy. The phagocytosed cells were digested and used for food or persisted against digestion and became permanent endosymbionts, acting as parasites, mutualists, or slaves (Cavalier-Smith, 1975). Successful endosymbiotic bacteria learned to induce their own internalization, reach the vacuole and persist against destruction. For this purpose, many intracellular bacteria evolved strategies to inhibit phagosomal maturation (Bozue and Johnson, 1996) or prevent oxygen radical production (Haas and Goebel, 1992). The benefits of vacuolar life for the internalized bacteria were being protected against environmental stresses while reaching plenty of nutrients needed for survival and proliferation (Garcia-del Portillo and Finlay, 1995; Finlay and Falkow, 1997; Greub and Raoult, 2004). Establishment of endosymbiotic bacteria within eukaryotic hosts led to maintenance of the vacuole and survival of both partners (Bianciotto et al., 1996). Accordingly, vacuole of eukaryotic cells represents a unique and sophisticated niche in which the endosymbiotic bacteria could survive and promote persistent association with the host cell (Cavalier-Smith, 2002).

In our previous studies we used microscopic and molecular biology methods to present evidence for the existence of non-culturable *Helicobacter pylori* inside the vacuole of *Candida* yeast (Siavoshi et al., 1998, 2005, 2013; Salmanian et al., 2008, 2012; Siavoshi and Saniee, 2014). Using anti-*H. pylori* egg yolk immunoglobulin Y (IgY-Hp) and western blotting, *H. pylori*-specific proteins; VacA, urease, peroxiredoxin, and thiol peroxidase were detected in the protein pool of *Candida* yeast, indicating that inside the yeast vacuole *H. pylori* is alive and expresses proteins (Saniee et al., 2013a). Fluorescent microscopy observations on *Candida* yeast cells treated with fluorescein isothiocyanate (FITC)-conjugated IgY-Hp, demonstrated the internalization of FITC-IgY-Hp into yeast cells and its specific binding with *H. pylori* cells, confirming the localization of *H. pylori* inside the yeast vacuole (Saniee et al., 2013b). Accordingly, yeast vacuole was proposed as a unique and specialized niche for accommodation of *H. pylori*, providing essential nutrients for its growth and multiplication (Saniee et al., 2013a,b; Siavoshi and Saniee, 2014). Intracellular existence of *H. pylori* has been reported in epithelial cells (Chu et al., 2010), macrophages and bone marrow-derived dendritic cells (Wang et al., 2009, 2010) and bacterial cells were observed within defined membrane-bound vacuoles (Segal et al., 1996; Dubois and Boren, 2007). It appears that *H. pylori* has evolved to equip itself for invading the eukaryotic cells and establishing in their vacuole (Dubois and Boren, 2007; Chu et al., 2010).

Reports describe occurrence of endosymbiotic bacteria in many eukaryotes, including protozoa, bivalves and insects (Douglas, 1994), Sponges (Erwin et al., 2012) and fungi (Scannerini and Bonfante, 1991). However, a considerable number of endosymbionts are non-culturable (Ruiz-Lozano and Bonfante, 1999; McFall-Ngai, 2008) and their intracellular localization and identification are possible by recruitment of microscopic and molecular biology methods (Bianciotto et al., 1996). In this regard, fluorescent dyes are ultra-sensitive markers that have been widely used in Live/Dead *Bac*Light staining and fluorescence *in situ* hybridization (FISH) methods for localization and identification of live but non-culturable bacteria inside eukaryotic cells (Bianciotto et al., 2000). Furthermore, egg yolk antibody (IgY) exhibits high affinity for its target antigen and strongly binds with cell plasma membrane due to positive charge and lipophilic nature (Kovacs-Nolan and Mine, 2012). Results of our previous study showed the internalization of FITC-IgY-Hp and its accumulation in the vacuole of *Candida* yeast, proposing that IgY when conjugated with a fluorescent dye could serve as a specific probe for localization and identification of intracellular *H. pylori* (Saniee et al., 2013b).

Endocytosis is a general mechanism by which eukaryotic cells internalize extracellular molecules through the formation of vesicles from the plasma membrane. The endocytosed particles internalize in a free state or while bound to a specific surface receptor. Once internalized by endocytosis, the cargo passes first through early endosome and next late endosome (Prescianotto-Baschong and Riezman, 2002) which fuses with vacuole and releases its contents (Hurley and Emr, 2006). The process of endocytosis is energy- and temperature-dependent and can be impaired by oxidative stress or incubation at low temperature. It is also time-dependent; the half-time for internalization has been estimated as 2–5 min (Pearse and Bretscher, 1981; Steinman et al., 1983). Endocytosis has been widely studied in yeast describing internalization of fluorescent dyes; FM4-64 (Vida and Emr, 1995) and lucifer yellow (Riezman, 1985) and nano gold particles (Prescianotto-Baschong and Riezman, 2002). In this study fluorescent microscopy was used to examine the uptake of FITC-IgY-Hp by *Candida* yeast cells, at different time intervals and its accumulation in the vacuole. Endocytosis inhibitors; low temperature, H_2_O_2_ or acetic acid were recruited to assess whether internalization of FITC-IgY-Hp into yeast cells is a vital phenomenon and follows the endocytosis pathway.

## MATERIALS AND METHODS

### YEAST STRAINS

Two gastric yeasts (G2 and G5) which were isolated from gastric biopsy cultures of two *H. pylori*-positive patients were used in this study. G2 was isolated from a 54 years old male and G5 from a 68 years old male. Patients were referred to Shariati hospital for endoscopy and diagnosed as having peptic ulcer. Informed consent was signed by the patients and the study was approved by research ethics committee of Tehran University of Medical Sciences. The primary identification of G2 and G5 yeasts as *Candida albicans* was according to microscopic morphology and production of green colonies on Chromagar (Odds and Bernaerts, 1994). Molecular identification of G2 and G5 yeast was performed by amplification of *Candida-*specific Topoisomerase II gene, using nested-PCR. Two sets of primers were used; CDF28 (GGTGWMGDAAYGGDTWYGGYGC), CDR148 (CCRTCNTGATCYTGATCBGYCAT) and CABF59 (TTGAACATCTCCAGTTTCAAAGGT), CADBR125 (AGCTA-AATTCATAGCAGAAAGC). PCR was performed according to Kanbe et al. (2002). Size of amplified product was determined as 637 bp by electrophoresis, using 100-bp molecular marker, confirming the identity of yeasts as *C. albicans.*

### PRODUCTION OF FITC-LABELED IgY-Hp

IgY-Hp was produced (Saniee et al., 2013a) and FITC-labeled (Wisdom, 2005; Saniee et al., 2013b) as previously described. Briefly, IgY-Hp was raised in hens by immunization with whole cell lysate of *H. pylori* and extracted from egg yolk according to Nikbakht Brujeni et al. (2011). FITC-IgY-Hp was prepared by adding FITC solution to the antibody and removing unbound FITC using Sephadex G25 column (Saniee et al., 2013b).

### CULTIVATION OF YEASTS

For time and inhibition assays, fresh culture of G2 and G5 yeasts on YGC (yeast extract-glucose-chloramphenicol) agar was inoculated into a home-made broth containing 5 g/L yeast extract (Pronadisa, France), 20 g/L *N*-acetylglucosamine (Sigma, USA), supplemented with equal volume of fetal bovine serum (Invitrogen, USA). Cultures were grown at 37°C for 24 h, washed twice and re-suspended in phosphate buffered-saline, PBS (0.5X).

### INTERNALIZATION ASSAYS

#### Time assay

G2 and G5 yeasts were used in this part of study. The intracellular occurrence of *H. pylori* inside the G2 yeast was determined by light and fluorescent microscopy as well as detection of *H. pylori*-specific genes and proteins in the whole extract of yeast cells. G5 yeast in which *H. pylori*-specific genes were not detected by PCR was used as a control (Saniee et al., 2013a,b). A volume of 100 μl of yeast cells in PBS was mixed with 20 μl of 1:5 dilution of FITC-IgY-Hp and 5 μl of evans blue solution (0.01% in PBS). Yeasts were incubated in dark at 37°C while shaking (200 rpm). Internalization of FITC-IgY-Hp into yeast cells was monitored in different time intervals, using fluorescent microscopy. Ten-microliter volumes of treated yeasts were taken after 0–5 min, 15 min, 30 min, 1, 1.5, 2, 3, 4, and 5 h, washed once with distilled water, re-suspended in PBS, spotted on the glass slide and air-dried. Dried spots were covered first with mounting oil (Invitrogen, USA) and next with coverslip. Nail polish was used for fixing the coverslip in place. Immersion oil was used for observing samples by fluorescent microscopy.

#### Low temperature assay

To examine the impact of low temperature on the endocytosis, a 100-μl volume of G2 yeast cells in PBS was mixed with 20 μl of 1:5 dilution of FITC-IgY-Hp and 5 μl of evans blue solution (0.01% in PBS) and immediately transferred to 4°C where it remained for 1 h while shaking manually every 10 min. Yeast cells were washed with ice-cold water. A 10-μl volume of yeast cells was spotted on pre-cold slide, air-dried on ice and kept cold until observed by fluorescent microscope.

#### Inhibition assay

To examine the inhibitory effect of H_2_O_2_ or acetic acid on internalization of FITC-IgY-Hp into G2 yeast cells, a 200-μl volume of yeast cells in PBS (0.5X) was treated with 10 mM of H_2_O_2_ or 140 mM of acetic acid and incubated at 37°C for 1 h with continuous shaking (200 rpm). To examine the possibility of negative effect of H_2_O_2_ or acetic acid on FITC-IgY-Hp or G2 yeast cells, at the end of incubation, each sample was divided into two 100-μl aliquots (tubes A and B). In tubes A, inhibitors remained in yeast samples. In tubes B, H_2_O_2_ or acetic acid was removed by harvesting and re-suspending the cells in 100 μl of PBS. FITC-conjugated IgY-Hp and evans blue solution were added to tubes A and B as mentioned above. Samples were incubated in dark at 37°C while shaking (200 rpm) and examined at different time intervals (0–5 min, 15 min, 30 min, 1, and 2 h) by fluorescent microscope.

To examine the viability of yeast cells after being treated with H_2_O_2_ or acetic acid, a 10-μl volume of yeast cells from tubes A and B was cultured on YGC agar after each time interval and incubated at 37°C.

## RESULTS

### EFFECT OF TIME ON INTERNALIZATION

**FIGURE 1:**
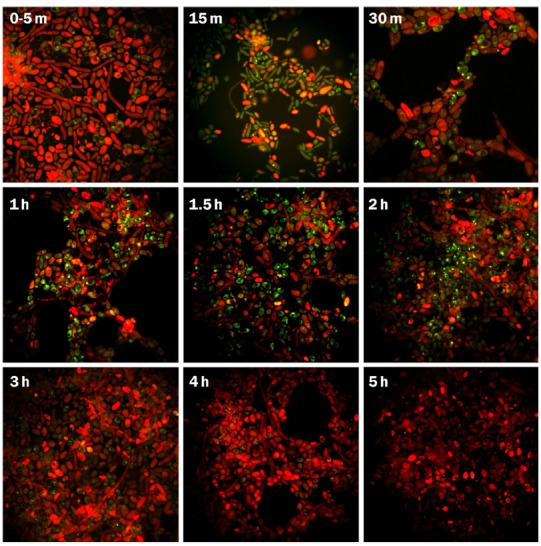
**Fluorescent microscopy of FITC-IgY-Hp-treated G2 yeast cells at different time intervals.** Fluorescent microscopy photographs show the internalization of FITC-IgY-Hp in 5–10% of yeast cells within 0–5 min, increased to 20–40% within 30 min to 1 h and reached >70% before 2 h. The green fluorescent-labeled *H. pylori* cells remained visible up to 3 h and fainted within 4–5 h when yeast cells appeared completely red, due to evans blue, with dark vacuole (magnification × 1000). m, minutes; h, hours.

**FIGURE 2:**
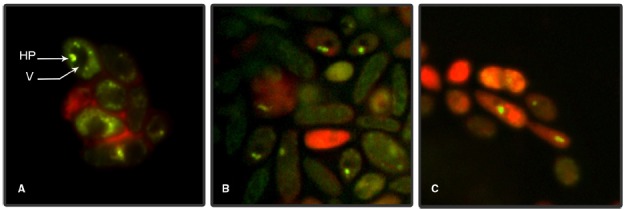
**Enlarged fluorescent microscopy photographs of G2 yeast cells.** Threefold enlarged yeast cells with original × 1000 magnification, showing green fluorescent spots in the yeast vacuole after 2 h incubation with FITC-IgY-Hp. **(A)** *H. pylori* (HP) in the vacuole (V) of yeast, **(B)** *H. pylori* in the vacuole of yeast, **(C)** *H. pylori* in the vacuole of a yeast cell and its bud.

**FIGURE 3:**
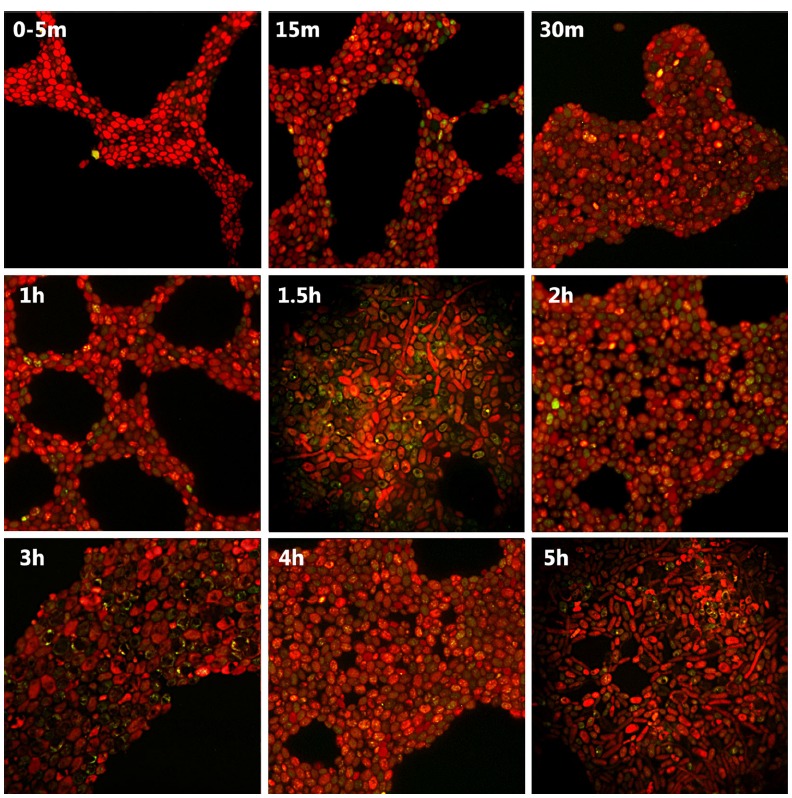
**Fluorescent microscopy of FITC-IgY-Hp-treated G5 yeast cells at different time intervals.** Fluorescent microscopy photographs show no regular pattern of increase in internalized FITC-IgY-Hp by time (up to 3 h) and faint within 4–5 h (magnification × 1000).

Fluorescent microscopy observations showed the internalization of FITC-IgY-Hp into G2 yeast cells and its accumulation in the vacuoles due to binding with *H. pylori* antigens. Internalization initiated immediately within 0–5 min and green fluorescent spots were observed in 5–10% of yeast cells. After 30 min–1 h, 20–40% of cells showed green fluorescent spots in their vacuoles. The number of yeast cells with green fluorescent spots reached >70% before 2 h. The green fluorescent-labeled *H. pylori* cells inside the yeast vacuole remained shining for 3 h and then started fainting. After 4–5 h, green fluorescent spots became invisible and yeast cells appeared completely red, due to evans blue, with dark vacuole (Figure 1). Threefold enlargement of microscopic photographs with original × 1000 magnification showed the specific interaction of FITC-IgY-Hp with *H. pylori* cells in the yeast vacuole, after 2 h incubation with labeled antibody (Figures 2A–C). Fluorescent microscopy observations of G5 yeast in different time intervals did not show the same pattern as G2 yeast. Green spots were observed in only few yeasts with less fluorescent intensity and persistence (Figure 3).

### EFFECT OF LOW TEMPERATURE, H_2_O_2_ OR ACETIC ACID ON INTERNALIZATION

**FIGURE 4:**
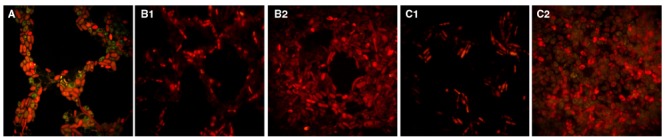
**Effect of endocytosis inhibitors on internalization of FITC-IgY-Hp into G2 yeast cells.** Fluorescent microscopy photographs show that yeast cells inhibited by low temperature (4°C), H_2_O_2_ (10 mM) and acetic acid (140 mM) did not accumulate green fluorescent antibody and appeared red. **(A)** The inhibitory effect of low temperature. **(B1)** Exposure (2 h) to FITC-IgY-Hp along with H_2_O_2_; tube A. **(B2)** Incubation (2 h) with FITC-IgY-Hp after H_2_O_2_ removal; tube B. **(C1)** Exposure (2 h) to FITC-IgY-Hp along with acetic acid; tube A. **(C2)** Incubation (2 h) with FITC-IgY-Hp after acetic acid removal; tube B (magnification × 1000).

Fluorescent microscopy of FITC-IgY-Hp-treated G2 yeast, incubated at 4°C for 1 h showed inhibitory effect of low temperature on internalization of labeled-antibody. Yeast cells did not accumulate green fluorescent antibody and appeared dark (Figure 4A). Fluorescent microscopy showed similar results of the inhibitory effect of H_2_O_2_ or acetic acid on the internalization of FITC-IgY-Hp into yeast cells. Fluorescent microscopy photographs showed that in tubes B (inhibitors removed), G2 yeast cells were clearly visible and red with dark vacuoles, without FITC-IgY-Hp accumulation (Figures 4B2,C2). In tubes A (inhibitors present) yeast cells did not accumulate FITC-IgY-Hp and showed altered morphology that could be the result of long exposure to inhibitors (Figures 4B1,C1). These results indicated that 1 h was sufficient for effective inhibition of FITC-IgY-Hp internalization by H_2_O_2_ or acetic acid. Inhibitory effect persisted in yeast cells even after 2 h of inhibitors removal. Cultures of G2 yeast on YGC were positive and colonies were observed after 24 h.

## DISCUSSION

IgY has been recruited as a powerful tool for diagnostic purposes. It is a positively-charged and lipophilic antibody with a molecular mass of ∼180 kDa, slightly larger than mammalian IgG, 150 kDa (Kovacs-Nolan and Mine, 2012). IgY-Hp strongly reacts with *H. pylori*-specific antigens and can be recruited for effective inhibition or detection of *H. pylori* (Shin et al., 2002). In our previous studies, strong interaction of IgY-Hp and FITC-IgY-Hp with *H. pylori* antigens, present in the vacuole of yeast, revealed the occurrence of live *H. pylori* cells inside the *Candida* yeast (Saniee et al., 2013a,b).

Results of this study showed that FITC-IgY-Hp could bind with negatively-charged lipid on yeast cell plasma membrane and internalize through endocytotic route in a time-dependent fashion. Internalization of labeled antibody which was determined by visualizing green fluorescent spots in G2 yeast vacuole, initiated immediately (0–5 min) in 5–10% of yeast cells, increased to 20–40% within 30 min–1 h and reached >70% before 2 h. Fainting started before 3 h possibly due to quenching of FITC-IgY-Hp at acidic pH of the yeast vacuole (Clarke et al., 2010). Comparison of photographs of G2 and G5 yeast especially within 1–2 h, showed that the frequency of green fluorescent spots was much higher in G2 yeast than G5 yeast. Furthermore the intensity and persistence of fluorescent spots in G2 yeast was higher than in G5 yeast. This could be due to strong interaction of FITC-IgY-Hp with *H. pylori*-specific antigens in G2 yeast. In contrast, in G5 yeast without *H. pylori* few fluorescent spots with less intensity and persistence were observed. These results show that although FITC-IgY-Hp uptake occurred through endocytotic pathway, the fate of labeled antibody was determined by its specific binding with *H. pylori* antigens, otherwise it would be denatured (Lobo et al., 2004) or quenched in acidic pH of yeast vacuole, in a short time. Internalization of FITC-IgY-Hp was inhibited at cold or upon treatment of yeast cells with endocytosis inhibitors; H_2_O_2_ or acetic acid. Inhibition of endocytosis was determined when treated yeast cells did not show accumulation of green fluorescent IgY-Hp and vacuoles appeared dark. Comparison of photographs from yeasts in tubes A and B showed that H_2_O_2_ or acetic acid did not have a negative effect on FITC-IgY-Hp. However, long exposure of yeast cells to endocytosis inhibitors led to altered morphology of yeast cells. Endocytosis of FITC-IgY-Hp did not occur in yeasts of tubes B, even 2 h after removal of inhibitors. Moreover, yeast cells in tubes A and B remained viable and produced colonies after 24 h.

Fluorescent microscopy studies on the endocytosis of FM4-64, a lipophilic fluorescent dye, in *Saccharomyces cerevisiae* revealed that the dye initially stained the plasma membrane and finally reached the vacuolar membrane after 1 h (Vida and Emr, 1995). However, internalization of dye was impaired when yeasts were kept at cold temperature (Huckaba et al., 2004) or exposed to 1–3 mM H_2_O_2_ or 80–140 mM acetic acid for 1 h (Pereira et al., 2012). Fluorescence microscopy was also used to study *C. albicans* cells exposed to a sub-inhibitory concentration (5 μg) of apolipoprotein-derived ApoEdpL-W-Fluo, a labeled antifungal peptide, after different time intervals. Fluorescent peptide was bound to the cell surface and accumulated in the vacuole of 33% of cells within 30 min, 66–87% after 60 min and almost all the cells after 2 h. The ApoEdp-W-Fluo was co-localized with FM4-64, indicating that the target organelle was vacuole (Rossignol et al., 2011). A Fluorescent microscopy study on the internalization of fluorescent dye lucifer yellow into *S. cerevisiae* vacuole showed that endocytosis of lucifer yellow was dependent on time, temperature and energy (Dulic et al., 1991). Electron microscopy observations on the internalization of positively-charged nano-gold particles into *S. cerevisiae* endosome revealed that they strongly bound with negatively-charged lipids on the surface, endocytosed and reached the vacuole (Prescianotto-Baschong and Riezman, 1998). Accordingly, results of our study indicate that internalization of FITC-IgY-Hp into yeast cells was a vital phenomenon and followed the principle of endocytosis in yeast.

A number of immunologic and microscopic methods have been recruited for localization and identification of intracellular *H. pylori* in gastric biopsies (Andersen and Holck, 1990; Ko et al., 1999) and cultured cells (Semino-Mora et al., 2003; Necchi et al., 2007). In these methods, there was no barrier such as cell wall to internalization of antibodies. However, in the present study passage of antibody through the yeast cell wall was the critical step. Internalization of fluorescent-labeled antibodies into yeast cells has been used for recognizing and visualizing intracellular epitopes, especially components of the cytoskeleton (Pringle et al., 1990). The procedure involves fixation of cytoplasmic contents and permeabilization of the yeast cells with cell wall lytic enzyme (Hašek and Streiblová, 1996). Results of this study demonstrated that FITC-IgY-Hp with molecular mass of ∼180 kDa, could traverse the non-permeabilized cell wall of *Candida* yeast, internalize into yeast cell through endocytosis and accumulate within the vacuole in a short time. The pore size of the yeast *Cryptococcus neoformans* cell wall was measured by cryoporometry as 1–30 nm (Eisenman et al., 2005) and electron microscopy as 60–300 nm (Rodrigues et al., 2007). Furthermore, atomic force microscopy determined the pore size of *S. cerevisiae* cell wall as 200 nm that could increase to 400 nm under stressful conditions (de Souza Pereira and Geibel, 1999). Although, controversies exist about the size of molecules that can traverse the pores of yeast cell wall, it has been proposed that fungal cell walls are capable of import or export of vesicles that carry food supply, waste products or digestive enzymes. These vesicles are flexible lipid membranes that can compress and ferry across the cell wall. Accordingly, cell wall plays an important role in vesicular trafficking in yeast and the determining factor is the pore size of the cell wall (Casadevall et al., 2009).

Results of this study indicate that *Candida* yeast cell wall has pores large enough to let the labeled antibody to pass through, bind with plasma membrane, initiate endocytosis and finally accumulate within the vacuole where it strongly binds with *H. pylori* antigens. It appears that inside yeast vacuole *H. pylori* cells maintained their antigenic identity detectable by specific antibodies. Accordingly, it is proposed that endocytotic uptake of FITC-IgY-Hp could be used as an easy and specific tool for demonstrating the intracellular existence of *H. pylori* inside the yeast vacuole. Furthermore, IgY raised against other intracellular bacteria could be used for their localization and identification inside the yeast vacuole. Recruitment of fluorescent-labeled antibodies against different bacterial species might provide a powerful tool for differentiating and quantifying the members of the possible mixed bacterial population inside the vacuole of yeasts.

### Conflict of Interest Statement

The authors declare that the research was conducted in the absence of any commercial or financial relationships that could be construed as a potential conflict of interest.
